# Prevalence of concussion and adherence to return-to-play guidelines amongst male secondary school rugby and hockey players

**DOI:** 10.4102/sajp.v77i1.1477

**Published:** 2021-01-20

**Authors:** St. John Taft, Liezel Ennion

**Affiliations:** 1 Department of Physiotherapy, Faculty of Community and Health Sciences, University of the Western Cape, Cape Town, South Africa

**Keywords:** concussion, rugby, hockey, return-to-play, sports injury

## Abstract

**Background:**

Concussion injuries are common in contact sports. Young players can suffer life-threatening complications if concussion is not recognised and managed.

**Objectives:**

To determine the prevalence of concussion amongst secondary school rugby and hockey players and describe players’ knowledge and adherence to return-to-play guidelines.

**Method:**

A mixed-method approach included Phase A, which utilised a questionnaire completed by 221 players (*n* = 139 rugby; *n* = 82 hockey) between 13 and 18 years of age, and Phase B, which utilised three focus group discussions of 15 participants who had suffered a concussion.

**Results:**

The prevalence of concussion (*n* = 221) was 31.2% (*n* = 69). Of those, 71% (*n* = 49) were rugby players. Those who had suffered a concussion were more confident in identifying symptoms of concussion in themselves and others compared with those who did not suffer a concussion (*p* = 0.001), were more aware of return-to-play guidelines and more confident in their knowledge of concussion (*p* = 0.001). There were no differences between groups when identifying concussion symptoms. Of those who had a concussion, 30.4% (*n* = 21) adhered to return-to-play guidelines and followed graded return-to-play after their concussion. Explanations for non-adherence to return-to-play protocols included peer pressure, intrinsic motivation and ignorance.

**Conclusion:**

Nearly a third had suffered a concussion injury; having suffered a concussion, and awareness of return-to-play guidelines, did not guarantee adherence to return-to-play protocols. Peer pressure and intrinsic factors explained this lack of adherence.

**Clinical implications:**

Physiotherapists are often involved with the diagnosis and management of concussion injuries among rugby and hockey players. Understanding the prevalence and the reasons why young players do not adhere to the ‘return to play’ guidelines may inform preventative strategies.

## Introduction

Concussion is a common injury amongst participants in contact sports (Guilmette, Malia & McQuiggan 2007; Holtzhausen et al. 2002). The prevalence of concussion differs from study to study (Fraas et al. 2014; Holtzhausen et al. 2002; Shuttleworth-Edwards et al. 2008), but concussion is the third most common rugby injury in South Africa (Shuttleworth-Edwards et al. 2008). Recently, there has been increased interest amongst healthcare workers to investigate the diagnosis, treatment and outcomes of mild traumatic brain injuries such as concussion (Fraas et al. 2014). In the last 5 years, the World Health Organization (WHO) and the Center for Disease Control (CDC) have made considerable efforts to increase the public’s awareness of the dangers and risks associated with concussion (McCrea 2008). In response to this increase in awareness of concussion, the ‘safe return-to-play guidelines’ for concussion were developed and made public at an international conference on concussion held in Zurich in 2012 (McCrory et al. 2013).

Concussion is considered a mild traumatic brain injury, which can be life threatening when the effects are compounded. If players do not report an initial concussion, they might be at risk for further injury such as second impact syndrome (Patricios et al. 2010). Second impact syndrome occurs when the brain has not fully recovered from a concussion injury and suffers another blow. During the second blow, the brain swells further, cerebral blood flow increases and therefore brain pressure increases, which could lead to death (Cantu 2001). Late complications of a concussion can include post-concussion syndrome (Omalu et al. 2005). This syndrome occurs when symptoms of a concussion manifest for days to weeks post-initial concussion, and symptoms include headache, memory loss, mood swings and inability to sleep, which can be debilitating (Omalu et al. 2005). Chronic traumatic encephalopathy is another possible complication of concussion and the result of repeated long-term exposure to concussive blows. Each concussive blow exponentially increases the risk resulting in residual brain damage (Omalu et al. 2005). Depression later in life and early-onset mild cognitive impairment have also been linked to multiple concussions (Omalu et al. 2005).

The ‘return-to-play’ process started after the first concussion conference held in Vienna, where experts recommended that the concussed athlete follow a stepwise process to return to sport (Cantu 2006). The guidelines that are used to help reintegrate a concussed player back to contact sport were developed at the concussion conference and redefined at each subsequent conference until the latest one in Berlin in 2016 (McCrory et al. 2017). According to these guidelines, a player who has sustained a concussion should stop playing immediately and be removed from the field.

The concussed player should then rest (physically and cognitively) until asymptomatic. Once asymptomatic, the player starts the next phase, which is light aerobic exercise such as walking, cycling or swimming. Phase three entails sport-specific drills such as running drills with no head contact. Phase four requires non-contact training drills, so the player can join practices but not engage in contact. Phase five is a full-contact practice following medical clearance, and phase six is full match play. If at any point in the six phases, players feel any post-concussive symptoms, they must stop and return to the previous phase of recovery (McCrory et al. 2017). During the recovery of a concussion, it is also useful to do neuropsychological testing to help monitor symptoms, given that players have performed baseline testing before injury (Broglio, Macciocchi & Ferrara 2007).

The ‘return-to-play’ guidelines for concussion are relatively new and not well adhered to (Sye, Sullivan & McCrory 2006). In a study conducted with high school rugby players in New Zealand, only 50% of the participants were aware of the ‘return-to-play’ guidelines even though there is a policy in place for concussed athletes (Sye et al. 2006). In a more recent South African study, only 20% – 23% of amateur rugby players knew about the ‘return-to-play’ guidelines after a concussion injury (Walker 2015). Before returning to play (practice or competition), a concussed athlete needs to be cleared by a medical professional (SCAT 2013). However, only 22.3% (66/296) of players in the New Zealand study sought medical clearance before returning to practice or competition (Sye et al. 2006). In the South African study amongst rugby union players, 74% expressed that they would engage in practice before fully recovering from a concussion injury (Walker 2015).

There is a lack of data on the prevalence of concussion amongst young athletes who participate in contact sports such as rugby and hockey. Due to the lack of adherence to ‘return-to-play’ guidelines, coupled with the considerable medical risk associated with concussion, our study aimed to determine the prevalence of concussion and the adherence of the ‘return-to-play’ guidelines amongst a sample of secondary school, male rugby and hockey players in South Africa.

## Method

Our study was conducted at a secondary public boys’ school in South Africa. A mixed-method approach and an explanatory sequential design were utilised. Data were collected in two phases. Quantitative data were collected first, then analysed, and the findings explained in more detail through an explorative description, qualitative phase.

### Phase A: Quantitative data

A questionnaire was used to collect data. The questionnaire was reviewed by a sports physiotherapist and a sports physician to determine face and content validity. The questionnaire was then piloted by 10 rugby and hockey players and completed again a week later to determine test–retest reliability. No changes were suggested by the pilot participants; therefore, their responses were included in our main study. The questionnaire was divided into four sections: (1) biographical data, (2) past prevalence of concussion, (3) knowledge of concussion symptoms and (4) adherence to the return-to-play guidelines. In 2016, 240 players registered to play rugby and 176 players registered to play hockey at the secondary public boys’ school. A total of 350 players in grades 8 to 11 were conveniently invited to participate in our study during the school’s designated sport period for each grade. During the roll call for the different sporting codes, the first author explained the purpose of our study to all players and distributed the parental consent, information sheets and participant assent forms to interested individuals. The first author then collected the signed parental consent and assent forms from those who agreed to participate, during the following week’s sport period and distributed the questionnaires.

According to Yamane’s (1967) formula, 204 study participants are required for a representative sample. In our study, 221 participants consented to participate with a 63% (221/350) response rate. Quantitative data were analysed by assigning numerical codes to each variable and capturing the coded data into the statistical package for the social sciences (SPSS) version 24.0. Data were analysed descriptively for measures of central tendency and frequencies, and Fischer’s exact test was used to determine if there were any significant non-random differences in the ability of self-perceived confidence in identifying the symptoms of concussion between those who suffered a concussion and those who did not suffer a concussion. The alpha level was set at 95%.

### Phase B: Qualitative data

From the data collected in the quantitative phase, 46% (*n* = 102) of the players did not adhere to the ‘return-to-play’ guidelines. Focus group discussions were used to better understand and explain the underlying reasons for non-adherence to the guidelines following concussion injuries. Of the 69 players who sustained concussion injuries, a purposive sample of 24 players who had suffered a concussion and stated they did not adhere to the return-to-play guidelines were invited to participate in three focus groups. Of these 24 players, 15 consented to participate in one of three focus group discussions. Each discussion lasted approximately 45 min and was conducted by the first author in a convenient office at the secondary school. All participants signed a focus group confidentiality consent form before the commencement of the discussion. The discussion was started with the following question: ‘If you returned to sport before being cleared by a medical professional, why did you not seek medical clearance after your concussion?’ Probing questions were used to follow up on statements made during the discussion. The focus group was audio-recorded and then professionally transcribed. The transcribed qualitative data were analysed by using thematic content analysis through a process of deductive coding and categorising to derive emerging themes. The themes were then verified by an independent researcher to ensure confirmability. Member checking was carried out to ensure that the themes accurately represented the views of the participants.

### Ethical consideration

Ethics clearance to conduct this study was obtained from the University of the Western Cape’s Human and Social Sciences Research Ethics Committee. Permission to conduct research in a public school was obtained from the provincial Department of Education, and permission to collect data from learners was obtained from the principal of the school. Informed consent was obtained in writing, from parents of minors and the participants themselves. Ethical clearance was received on 07 September 2016 (Ethical Clearance number: HS16/5/33).

## Results

### Quantitative results

#### Biographical data

A total of 221 rugby and hockey players responded and completed the questionnaire. The average age of the participants was 15.32 years (standard deviation [SD] ± 1.35), ranging from 13 to 18 years. Three-quarters of the sample (75.1%) was between the age of 13 and 16 years. The majority (62.9%; *n* = 139) played rugby.

#### Past prevalence of concussion

The past prevalence of concussion was 31.4% (*n* = 69). Of those who suffered a concussion injury, 71% (*n* = 49) were rugby players. Overall, 37.3% (*n* = 26) of the players with concussion were single incidences, with 62.7% suffering more than one concussion. Rugby players suffered an average of 1.73 concussions per player ranging from one to five concussions. Hockey players suffered an average of 1.28 concussions per player with the maximum number being three over their playing careers. The maximum number of concussions sustained by a single participant (a rugby player) was five (Table 1).

**TABLE 1:** Prevalence of concussion by sport (*n* = 221).

Sport	Frequency		Mean number of concussions	Standard deviation	Range
	*n*	%			
Rugby	49	71	1.7347	1.05624	1–5
Hockey	18	26.1	1.2778	0.57451	1–3
Non-specified	2	2.9	-	-	-
**Total**	**69**	**100**	**1.6119**	**0.96852**	**1–5**

**FIGURE 1:**
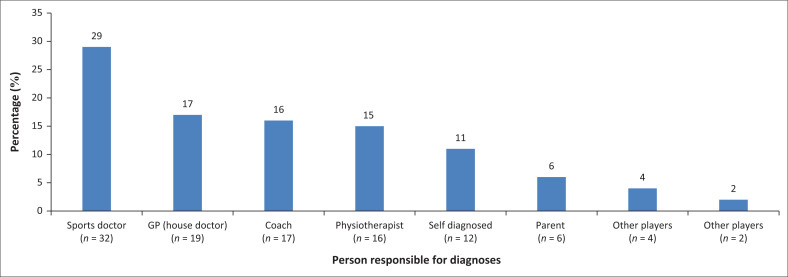
Person responsible for diagnosing concussion.

A total of 108 concussions were sustained, and the majority (61%, *n* = 66) were diagnosed by a health professional (a sports doctor, general practitioner or physiotherapist) (Figure 1). Similar percentages (16%, *n* = 17; 15%; *n* = 16) of diagnoses were made by the coach and a physiotherapist.

#### Perceived knowledge of concussion symptoms and the ‘return-to-play’ guidelines

Those who previously suffered a concussion had more *self-perceived confidence* in diagnosing (identifying symptoms) a concussion (*p =* 0.001) compared with those who had not suffered a concussion. There were no statistically significant differences in the knowledge of concussion symptoms between the two groups when actually identifying symptoms of concussion. However, those who previously suffered a concussion were significantly (*p* = 0.030) *more aware* of the ‘return-to-play’ protocols, compared with those who did not suffer a concussion. Unfortunately, having an awareness of the protocol did not guarantee adherence to it.

When participants were asked to tick the phrase that explained the ‘return-to-play guidelines’ after concussion, the most commonly incorrectly identified phrase was ‘return-to-play after a period of rest’ with 67.4% (*n* = 149) responses.

Fewer than half (48.2%) of the participants ticked ‘gradual return to sport activity once symptom free’. There was a statistically significant (*p* = 0.030) difference in the number of correct responses between those who had suffered a concussion and those who had not. Those who previously suffered a concussion had more knowledge about the guidelines than those who did not.

The majority of participants (93%; *n* = 205) were aware of the fact that a parent should not be responsible for clearing the player to participate again after concussion. A general practitioner (70.6%; *n* = 156) or sports physician (64.3%; *n* = 142) were amongst the most common choices selected.

#### Self-reported adherence to return-to-play guidelines

Only a third (30.4%; *n* = 21) of the participants who suffered a concussion followed a structured and graded ‘return-to-play’ protocol. Of those who suffered a concussion, 20.2% (*n* = 12) ‘did nothing’ as a form of treatment.

Of those who suffered a concussion, 25.4% rested from sports fewer than 10 days after suffering the concussion. Just over half (55.2%; *n* = 37) rested between 10 and 21 days. Similarly, approximately half (52.2%; *n* = 36) of the participants who suffered a concussion were cleared by a medical professional before they returned to sport.

## Qualitative results

The participants in the qualitative study were purposively selected based on their responses in the questionnaire, indicating that they did not adhere to ‘return-to-play’ guidelines.

Even though most of the participants agreed on the importance of being cleared by a medical professional before returning to play, the majority either ignored the return-to-play protocols that are in place or skipped a few steps in the protocol evidenced by the following quotes:

‘I somewhat do it depending on the extent of the injury, if it’s not that serious and they say maybe recovery time is less than a week or week maybe it’s not that important to rest that week’. (Participant 4, Male, Focus Group 3)

‘… personally it depends on how bad it is so for instance now I have a concussion from Saturday, but I feel like I could still play, personally I think I can go back maybe after resting on the weekend maybe two days …’ (Participant 3, Male, Focus Group 3)

When explaining their reasons for non-adherence to return-to-play protocols, three themes were deduced from the discussions, namely (1) peer pressure, (2) intrinsic motivation and (3) ignorance despite awareness of risk.

### Peer pressure

Participants who returned to sport earlier than what they were supposed to did it because of feelings of peer pressure or perceptions that their team was under pressure.

‘I remember it was a process like four weeks or something I returned like a little bit earlier I think it was to the fact that maybe it was just like pressure on the team or peer pressure or something like that.’ (Participant 2, Male, Focus Group 1)

‘I think maybe sometimes it could be your teammate as I was saying that they need you this weekend or something in that regard …’ (Participant 1, Male, Focus Group 3)

### Intrinsic motivation

The majority of participants in the focus groups mentioned that intrinsic factors contribute to them not wanting to report concussions or not follow the correct ‘return-to-play’ protocols. These include not wanting to let down the team, not wanting to get dropped from a team (especially if they have worked hard to get into that team), passion for the sport and school, what grade you are in, the team you play for or how late it is in the season.

‘I wanted to play as quick as possible.’ (Participant 4, Male, Focus Group 3)

‘I wanted to play, I needed to play.’ (Participant 2, Male, Focus Group 3)

‘… for me in terms of our last year of high school whether it being your last two or three games um you kind of want to finish off those last two games as you are leaving high school so for me that would stop me, obviously depending on my symptoms and if I was severely concussed.’ (Participant 6, Male, Focus Group 3)

‘I think knowing that if you get concussed you will probably get dropped a team and for Matrics your last year it is a hard thing if it is late in the season and you got to come back.’ (Participant 1, Male, Focus Group 3)

‘… when you love rugby you don’t want to just not play you know what I mean like if you get told you have a concussion then you know you out for about a month and a lot of people don’t want that to not play for their school.’ (Participant 4, Male, Focus Group 2)

Some of the participants mentioned that they would try and hide a concussion from a coach or parent because they were worried about not being able to participate.

‘… also the same sir I don’t want to its more myself trying to, my parents and coaches know if you injured they won’t let you play, it’s more like me telling myself not to go off now.’ (Participant 1, Male, Focus Group 1)

‘… also at the time you take your hit you might think you concussed you think you can hide the symptoms and all of that, so you can just carry on playing …’ (Participant 1, Male, Focus Group 2)

### Ignorance despite awareness of risk

Most participants knew and felt that it was important to be cleared by a medical professional before returning to play after suffering a concussion. This was evident when asked if they feel it is important to be cleared by a medical professional before returning to sport.

‘Yes, because if you take another knock without being ready to go play again it can be really bad. I have heard stories of people who can’t play rugby again.’ (Participant 5, Male, Focus Group 2)

‘Yes, it is important because at the end of the day it is your life on the line and I would rather be safe than die on the field.’ (Participant 5, Male, Focus Group 3)

However, despite knowing the dangers associated with concussion, some participants continued to ignore the guidelines.

‘I don’t (adhere to the guidelines) besides understanding what I am supposed to do or what is happening, I still feel that the need to play is more than the need to be safe for me.’ (Participant 2, Male, Focus Group 3)

## Discussion

The prevalence of concussion in this study was 31.4% (*n* = 69). There is a large variance of point and past prevalence of concussion ranging between 13% and 38% when considering national and international studies (Brown et al. 2012; Delahunty et al. 2015; Noakes & Du Plessis 1996). Before our study, only three studies had been undertaken on the prevalence or incidence of concussion in a South African high school rugby context. One determined the point prevalence in rugby union players in 1996 (Noakes & Du Plessis 1996), one the incidence across three different schools (Shuttleworth-Edwards et al. 2008) and one the prevalence of high school and amateur rugby players in 2017 (Viljoen et al. 2017). When comparing our prevalence to these earlier studies, it is much higher than the 21.5% point prevalence reported in one season in 1996 (Noakes & Du Plessis 1996) and the incidence (4% – 14%) reported in 2008 and is similar to the 33% prevalence that was reported amongst high school players in 2017.

The discrepancy with the 1996 and 2008 studies could be owing to the fact that because they were undertaken, the awareness of the symptoms and dangers of concussion may have increased. The fact that the prevalence of concussion is comparable to the figures reported in 2017 is a reason for concern. It seems that the ‘return-to-play’ guidelines and efforts to increase awareness of the dangers of concussion since the guidelines were introduced in 2012 have not contributed to decreasing the prevalence of multiple concussions in South Africa over the past 3 years. The prevalence in our study is higher than the prevalence of 19.4% that was reported in a similar Irish study conducted in 2015 (Delahunty et al. 2015).

The prevalence of concussions was significantly higher amongst rugby players compared with hockey players. Hockey is played with a hockey stick and ball, and no player-on-player contact is allowed. Concussions in rugby are mainly because of high player-on-player contact. More than 75% of concussions that were reported were as a result of player-on-player contact, and 15.5% of concussions happened from contact with the playing surface (Meehan, d’Hemecourt & Comstock 2010). In field hockey, however, concussions mainly occurred during player–equipment contact, resulting in 60.8% of concussion injuries with contact between ball and player equalling 37.3% and contact between stick and player equalling 23.5% (Marar et al. 2012). There are no other studies on the prevalence of concussion in South African hockey; however, a study based in American college field hockey stated that out of all the head, face and eye injuries, concussion counted for 42.8% of injuries sustained (Gardner 2015). More research should be performed into concussion amongst young hockey players because a substantial number of hockey players did report sustaining a concussion in our study.

Multiple concussions compound the risk for second impact syndrome and chronic traumatic encephalopathy (Cantu 2006; Patricios et al. 2010). Rugby players suffered more multiple concussions (1.73) compared with hockey players (1.27). Of the rugby players who suffered a concussion, 43% suffered more than one concussion with two participants reporting suffering five concussions. Multiple concussions seem to be common amongst rugby players. Similarly, an Irish study reported that 32.3% of professional rugby union players who suffered a concussion also suffered multiple concussions with the maximum number being four. The average number of concussions in the Irish study was 1.42 per player in the 2010–2011 rugby season (Fraas et al. 2014). Amongst three South African schools, the average number of self-reported concussions for rugby players was 2.3 (ranging from 0 to 7) (Shuttleworth-Edwards 2008).

If you suffer a second concussion, the force that is required to produce a concussive blow declines for every subsequent concussion that is received, and the brain takes longer to heal per concussion. A concussed athlete is four times more likely to suffer a second concussion after the first one, increasing the odds for re-injury (McBride 2012). A second concussion that occurs before the brain heals properly from the initial concussion could slow recovery or increase the likelihood of having long-term problems. Permanent brain damage, brain swelling or death is possible with repeated concussions (Centers for Disease Control and Prevention 1997, 2007).

Generally, the physiotherapist or team doctor is most likely to diagnose a concussion (Fraas et al. 2014). In our study, medical professionals were responsible for diagnosing 61% of all concussions. The concern is that 39% of the concussions were diagnosed by non-medical professionals, which could result in misdiagnosis and under-reporting of concussions in this setting. The reasons for not seeking medical advice are unclear. It could be because of the lack of availability of a medical professional working at the school or lack of medical insurance or finances to pay for medical care. However, Brown et al. (2012) reported that having a medical aid did not influence whether a concussed player would seek medical advice. Instead, it highlighted the parents’ or players’ perceived ‘necessity’ for it as the most common reason given for seeking professional advice. Sports coaches or parents might not have adequate knowledge of the symptoms of concussion, which might put players at risk for further brain damage or death.

The perceived confidence in diagnosing a concussion was not supported when the knowledge of symptoms of concussion was evaluated. To the authors’ knowledge, there is no other study reporting on self-perceived knowledge of concussion with which to compare our findings. However, it appears to be commonly known that players do self-diagnose concussions. Players commonly diagnose their own concussions based on knowledge gained through their personal experience or experience that they have seen from a friend or teammate (Chrisman, Quitiquit & Rivara 2013). This perceived confidence of players to diagnose themselves can put players at more risk of serious secondary complications.

Out of those who suffered a concussion injury, 30.4% (*n* = 21) of participants followed a graded ‘return-to-play’ protocol. Approximately, 20.2% (*n* = 14) of those who suffered a concussion injury indicated that they had received no treatment. Of the participants who had suffered a concussion, 53.7% were cleared by a medical professional before they returned to the sport. This shows poor adherence to the protocols that are in place. This is similar to a study that examined high school rugby players’ concussions, and out of the 62% of athletes who suffered a concussion, 20% did not report their concussions (Sye et al. 2006).

Some participants who returned to sport earlier than expected stated that peer pressure was a motivation for not following the graded return-to-play protocol. A number of intrinsic factors contributing to their not wanting to report concussions or not following the correct return-to-play protocols were raised in the focus group discussions. These included not wanting to let the team down, not wanting to get dropped from a team (especially if they had worked hard to get into that team), passion for the sport and school, what year they were in, the team they played for or how late it was in the season. Most of the participants knew and felt that it was important to be cleared by a medical professional before returning to play after suffering a concussion. However, this awareness of the protocol did not result in players adhering to the protocol.

Peer pressure, intrinsic motivation and ignorance despite awareness of risk were identified as reasons for not adhering to the return-to-play guidelines. Participants mentioned that their teammates place some pressure on them to play sooner, and some stated they did not want to be dropped from a team or want to miss their final year of school sport. This is similar to a study that showed that there are two types of pressures that have an impact on whether a player adheres to rehabilitation. One reason is social pressure, which could be from parents or peers, and the other being competitive pressure, which comes from the player having to compete for his spot in the team (Bloom et al. 2004; Niven 2007). In team sports, this pressure could be higher than that in individual sports (Bloom et al. 2004). With team sports, the sense of belonging and identity over and above the pressure from peers, parents or coaches could contribute to the lack of adherence of concussed athletes to the correct return-to-play protocols.

## Recommendations

Players, coaches, parents and medical staff need to be educated to be more aware of the risks associated with concussion and ensure that players are fully recovered from their first concussion before they return to sport. If a player does sustain a second concussion, they then need to be made aware that it may take longer to heal than the first concussion and that the protocol in place is a generalised protocol for a single concussion. Proper concussion history needs to be taken into consideration by sports doctors or medical professionals when players present with multiple concussions.

Parents and coaches, in general, should be educated on the symptoms of concussion, the importance of concussion rehabilitation and the potential complications of not following a ‘graded return to sport programme’. Parents and coaches will then be able to identify a concussion and make appropriate referrals to medical professionals for further in-depth assessments. By doing so, parents and coaches may reduce the risk of further complications.

## Limitations

At the time of data collection, the authors were unable to access the Rosenbaum Concussion Knowledge and Attitude Survey that was developed in 2010. However, measures were taken to establish the face and content validity and reliability of the questionnaire. Self-report of concussion injury is thus a limitation of our study.

## Conclusion

Nearly a third of our study sample had suffered concussions. Not all concussions are being diagnosed by medical professionals, which could lead to misdiagnosis of concussion and therefore the mismanagement of the ‘return-to-play’ guidelines. Peer pressure and intrinsic factors contributed to non-adherence to the ‘return-to-play guidelines of concussion’. The authors recommend that all players, coaches and parents are trained to be able to identify the signs and symptoms of concussion and the ‘return-to-play guidelines’. Non-medical professionals who are close to young athletes should be educated on how to identify a concussion and inform them who the best people are to refer them for further assessment. Rehabilitation services should be easily accessible to concussed athletes in order for them to gain medical clearance before returning to sport.
